# Investigating a foodborne illness outbreak at a private girls’ school in Mashonaland East Province, Zimbabwe, 2015

**DOI:** 10.11604/pamj.supp.2018.30.1.15271

**Published:** 2018-05-18

**Authors:** Tsitsi Juru, Nsiande Lema, Daniel Chirundu, Rayyan Muhammad Garba, Joseph Asamoah rimpong

**Affiliations:** 1 Zimbabwe Field Epidemiology Training Program, Harare, Zimbabwe; 2 Tanzania Field Epidemiology training program, Dar es Salaam, Tanzania; 3 Kadoma City Health Department, Kadoma, Zimbabwe; 4 Department of Community Medicine, Aminu Kano Teaching Hospital, Nigeria; 5 African Field Epidemiology Network, Accra, Ghana

**Keywords:** Outbreak investigation, foodborne illness, Zimbabwe

## Abstract

Globally, it is estimated that foodborne-associated illness accounts for 2.2 million deaths. This is caused by contamination of food with toxins, parasites, bacteria or viruses that can lead to increased levels of morbidity and mortality. Although steps to conducting an outbreak investigation have been outlined in most epidemiology textbooks, identifying the causative agent for a foodborne illness outbreak can be complex based on the setting. In view of that, this case study was developed based on a foodborne illness outbreak at agirls’ boarding school to model the steps of an investigation. This case study will reinforce skills and theoretical knowledge attained by public health trainees, to be able to build competences in foodborne outbreak investigation. The target audiences are intermediate and advanced public health trainees. Estimated time of facilitation is 3 hours with a class size of 10- 20 students.

## How to use this Study

**General instructions:** ideally, 1 to 2 instructors facilitate the case study for 10 to 20 students in a classroom or conference room. The instructor should direct participants to read a paragraph out loud, going around the room to give each participant a chance to read. When the participant reads a question, the instructor directs all participants to perform calculations, construct graphs, or engage in discussions. The instructor may split the class to play different roles or take different sides in answering a question. As a result, participants learn from each other, not just from the instructors.

**Audience:** postgraduate students in public healthand others who may be interested.

**Prerequisite:** lectures on outbreak investigation, study design and measures of association and impact.

**Materials needed:** laptop with Microsoft Excel or graph paper, flipchart or white board with markers, scientific calculator.

**Level of training:** advanced-outbreak investigation.

**Time required:** approximately 3 hours.

**Language:** english.

## Competing interest

The authors declare no competing interest.

